# Development and Application of Cancer Stem Cell-Targeted Vaccine in Cancer Immunotherapy

**DOI:** 10.4172/2157-7560.1000371

**Published:** 2017-10-31

**Authors:** Ming Lin, Alfred E. Chang, Max Wicha, Qiao Li, Shiang Huang

**Affiliations:** 1 University of Michigan Comprehensive Cancer Center, Ann Arbor, MI 48109, USA; 2 Department of Pediatrics, Union Hospital, Tongji Medical College, Huazhong University of Science and Technology, Wuhan 430022, China; 3 Union Hospital, Tongji Medical College, Huazhong University of Science and Technology, Wuhan, 430022, China

**Keywords:** Cancer stem cell, Dendritic cell, Vaccine, PD-L1, CCR10, Immunotherapy

## Short Communication

Accumulating evidence shows that tumours contain a distinct subpopulation of cancer stem cells (CSCs) which are capable of self-renewal, differentiation, and tumour-initiation[1,2]. Previous studies have demonstrated that cancer stem cells are relatively resistant to chemo-and radio-therapies[3,4], and play an essential role in tumour recurrence and metastasis. Since multiple human malignancies are associated with quantitative and qualitative deficiencies in the immune system, immunotherapies are potentially effective for cancer treatment. Vaccination is believed to have advantages over traditional treatments due to its potential to possibly eradicate the micrometastases that tend to linger after standard treatments. However, to date, experimental evidence remains limited on direct targeting of cancer stem cells by vaccination-induced immunotherapy.

In recent years, multiple studies have demonstrated that aldehyde dehydrogenase (ALDH) can serve as a specific marker for cancer stem cells in a variety of cancers[5–10]. We first characterized the tumourigenicity and stemness of ALDH(high) enriched cancer cells in immunocompetent animal models, and developed a dendritic-cell based cancer stem cell vaccine (CSC-DC) in murine melanoma (D5) and murine squamous cell carcinoma (SCC7) tumour models[10]. DC-based CSC vaccine is generated by using dendritic cells harvested from syngeneic mice and pulsing them with ALDH(high) cancer stem cells lysates. CSC-DC vaccine-primed CTLs and antibodies can then recognize and attack the CSCs. Our early study clearly showed that the CSC-DC vaccination could confer significant protective immunity against tumour cell challenges by selectively targeting cancer stem cells[10].

For a vaccine to be clinically relevant, it needs to be examined in the therapeutic models. Since solid tumours harbor only a small number of cancer stem cells[11,12], therapies that combine cancer stem cell vaccination with traditional treatments such as surgery, chemotherapy, and/or radiotherapy may optimize the therapeutic effectiveness against cancer. We postulate that the initial therapy of established tumours with traditional treatments may result in tumour shrinkage, and CSC-targeted vaccination may eliminate microscopic residual disease, resulting in reduced/delayed local tumour recurrence and distant metastasis. In two recent studies, we administrated DC-based cancer stem cell vaccination after localized radiotherapy of established tumours[13] or after surgical excision of the established tumours[14] in immunocompetent mouse models. CSC-DC vaccination significantly reduced local tumour relapse, inhibited spontaneous lung metastases, and prolonged host survival. No significant toxicities or adverse events were observed in these animal studies. However, to translate these findings into clinical, several critical problems remain to be solved. For example, the identification of any potential CSC antigen(s) recognized by CSC-DC vaccine-primed T cells and antibodies has yet to be characterized, and the mechanisms that are involved in CSC-DC vaccine-mediated therapeutic efficacy have to be fully defined.

To explore the mechanism (s) underlying the antitumour effect of the CSC-DC vaccine, we assessed the systemic immune responses elicited by DC-based cancer stem cell vaccination. Our results showed that CSC-DC vaccination conferred host CSC-specific CTL activity and antibody responses, resulting in significantly reduced population of ALDH(high) CSCs in treated tumours[14]. In addition, new vaccine adjuvants for cancer immunotherapy are being actively developed as an effort to augment vaccine-induced immune response against tumour-specific antigens in several studies[15–17]. Investigation and application of novel adjuvant may enhance the therapeutic efficacy of CSC-DC vaccine.

Programmed death ligand 1 (PD-L1) is a critical molecule found on the surface of tumour cells. PD-L1 molecules render tumour-reactive T cell tolerance to tumour cells by binding to programmed death-1 (PD-1) expressed on activated T cells[18,19]. Anti-PD-L1, acting as an immune checkpoint inhibitor, can block PD-1/PD-L1 interaction-dependent immune suppression. So far, several checkpoint inhibitors have been approved by the U.S. Food and Drug Administration. In a clinical trial, Gettinger et al. reported that anti–PD-1 antibody Nivolumab brought about durable responses and enhanced survival rates in patients with heavily pretreated non-small cell lung cancer (NSCLC)[20]. In our pre-clinical study, we surgically removed head and neck SCC7 subcutaneous tumours followed by ALDH(high) SCC7 CSC-DC vaccines. In addition, anti-PD-L1 was intraperitoneally (i.p.) injected either alone or with the ALDH(high) SCC7 CSC-DC vaccine. Results showed that ALDH(high) SCC7 CSC-DC vaccination plus anti-PD-L1 administration significantly inhibited tumour relapse and prolonged animal survival as compared with either treatment alone[14]. These experiments demonstrated that immunologically targeting cancer stem cells, while simultaneously blocking PD-1/PD-L1–mediated immune suppression, could significantly enhance the efficacy of cancer immunotherapies.

In spite of the fact that solid tumours account for the major cancer burden, over 90% of mortality in cancer patients were attributed to the subsequent spread of cancer cells to distant tissues[21]. CSCs mediate tumour metastasis, nevertheless, evidence of cancer immunotherapies targeting the CSCs is limited. We evaluated the therapeutic efficacy of the CSC-DC vaccine in the setting of micrometastatic disease, utilizing the highly metastatic D5 mouse melanoma model. However, metastasis is a multi-step process, and epithelial-mesenchymal transition (EMT) is also recognized as a relevant process during the progression of carcinomas towards metastatic disease. In a phase I clinical trial, Heery et al. described the ability of a poxviral-based vaccine to activate T cells specific against brachyury, a transcription factor known to participate in the processes of EMT[22]. In contrast to our study which targets CSC-associated antigens, this vaccine targets the process of mesenchymalization to minimize tumour dissemination. Accumulating evidence indicated that CSCs also express EMT markers, and more importantly, induction of EMT in transformed epithelial cells promotes the generation of CSCs[23–27]. In our study, the therapeutic efficacy of CSC-DC vaccine was associated with significantly inhibited metastasis of the subcutaneous tumour to the lung[14].

The involvement of chemokines and their receptors in cancer development, particularly metastasis, has been established over the last decade[28]. We examined the expression of several chemokine receptors on the highly metastatic D5 cells, and found that ALDH(high) D5 CSC-DC vaccination treatment significantly decreased the expression of CCR10 on D5 tumour cells compared with other treatment groups. On the other hand, PCR analyses showed that ALDH(high) D5 CSC-DC vaccine significantly reduced the mRNA levels of CCR10 ligands, e.g. CCL27 and CCL28, in lung tissues harvested from the D5-bearing hosts. More importantly, we found that while the expression of CCR10 was significantly higher on D5 ALDH(high) CSCs than on ALDH(low) non-CSCs, ALDH(high) D5 CSC-DC vaccine significantly decreased the expression of CCR10 on both ALDH(high) D5 cells and ALDH(low) D5 cells[14]. However, the molecular and biochemical signaling pathways by which CSC-DC vaccination induces down-regulation of CCR10 on cancer cells, particularly on cancer stem cells, and down-regulation of CCL27 and CCL28 in metastatic target organs have yet to be identified.

Together, these experiments have offered direct evidence that CSC-DC vaccine could induce significant antitumour effect by immunologically targeting cancer stem cells. Combination of CSC-DC vaccine strategy with traditional therapy, e.g. surgery, chemotherapy and/or radiation therapy; simultaneously blockage of immune checkpoint–mediated immune suppression; investigation and application of novel adjuvant, and understanding of the mechanism(s) underlining the CSC-based vaccine represent critical respects in future research and application of CSC-targeted cancer immunotherapy.
